# Reproduction on the Rocks: Life History of a Freshwater Macrobioeroding Bivalve

**DOI:** 10.1002/ece3.74329

**Published:** 2026-09-13

**Authors:** J. Reuben Shipway, Daniel L. Distel

**Affiliations:** ^1^ The International Marine Wood Borer Network Birmingham UK; ^2^ Naked Clam Limited Cambridge UK; ^3^ Ocean Genome Legacy Center Northeastern University Nahant Massachusetts USA

**Keywords:** broadcast spawning, drift paradox, ecosystem engineer, freshwater ecology, shipworms

## Abstract

Macrobioerosion, the excavation and removal of consolidated mineral substrates by macrofauna, is well established in marine systems, where macrobioeroders drive carbonate cycling, sediment production and habitat formation. In freshwater ecosystems, however, it has been documented in only a small number of invertebrate taxa and remains a poorly resolved ecological process. Among these, the teredinid shipworm *Lithoredo abatanica* represents a remarkable departure from the wood‐boring ecology of its family, having evolved to excavate and ingest limestone in fresh water. Despite this remarkable ecological transition, its reproductive biology and life history remain unknown. Here, we investigate the reproductive mode and life history strategy of this species using population size structure, in situ observations of siphonal morphology, and sperm morphometrics. We show that *L. abatanica* reaches exceptional dimensions, with measured body lengths exceeding 100 mm, one intact empty burrow exceeding 200 mm and in‐water observations indicating burrows possibly exceeding 500 mm, establishing it as the largest known freshwater macrobioeroder. Its large size and dense aggregations indicate considerable capacity for local rock breakdown and habitat modification within the Abatan River system (Bohol, Philippines). Recently settled juveniles (< 10 mm) alongside reproductively mature individuals indicate ongoing recruitment. The morphology of the siphons and calcareous tube appears to preclude direct sperm transfer via pseudocopulation, while sperm morphometrics are consistent with external fertilisation. Together, these findings indicate that *L. abatanica* reproduces via broadcast spawning with external fertilisation and likely possesses a planktotrophic larval phase. This raises a fundamental question: how does a broadcast‐spawning species with planktotrophic larvae maintain populations up to 15 km upstream in a flowing freshwater river subject to persistent downstream advection? By resolving the life history of the largest known freshwater macrobioeroder, this study provides critical insight into the persistence, dispersal and ecological role of a globally unique riverine ecosystem engineer.

## 1 Introduction

Animals that excavate freshwater substrates can profoundly alter physical habitats. Crayfish disturb and redistribute riverbed sediments, predatory stonefly larvae remove sand from gravel interstices while foraging, and freshwater mussels can compress and stabilise bed sediments (Mason and Sanders 2021). Macrobioerosion is a more specialised process involving the active excavation, boring or ingestive penetration of consolidated mineral substrates by macrofauna, resulting in the removal of rock and production of biogenic traces (Bolotov et al. 2018). Although macrobioeroders are major agents of carbonate cycling, sediment production and habitat formation in marine ecosystems (Davidson et al. 2018), only six named freshwater macroinvertebrates have been documented excavating lithified rock: the shipworm *Lithoredo abatanica*, the pholadid bivalve *Lignopholas fluminalis*, the unionid mussel *Balwantia soleniformis*, the mayflies *Languidipes lithophagus* and *Povilla adusta*, and the chironomid *Axarus festivus* (Godwin‐Austen 1919; Annandale 1919; Bidwell 1979; Ferrington 1992; Bolotov et al. 2018, 2022, 2024; Shipway, Altamia, et al. 2019; Shipway, Rosenberg, et al. 2019). Whether this limited record reflects genuine ecological rarity, incomplete investigation of submerged freshwater hardgrounds, or both remains unresolved. Among these taxa, the reproductive biology and larval ecology of *L. abatanica* remain unknown. Here, we investigate how this freshwater rock‐boring shipworm reproduces and consider the implications for its persistence and ecological role in a flowing river.

Shipworms (Teredinidae) are highly specialised bivalves best known for their ability to bore into and consume wood, where they play a central role in biodegradation, nutrient cycling and habitat modification across marine and estuarine ecosystems (Cragg et al. 2015; Hendy et al. 2022; Dickson et al. 2025). This xylotrepetic and xylotrophic lifestyle has driven the evolution of a distinctive suite of anatomical and physiological adaptations, including reduced anterior valves modified for boring, a vermiform body encased in a calcareous tube, and bacterial symbioses that facilitate the digestion of lignocellulosic material (O'Connor et al. 2014; Stravoravdis et al. 2021; Goodell et al. 2024). The main body, excluding the siphons, extends essentially from the blind anterior excavation face to the burrow aperture, with the shell valves positioned at the former and the siphonal bases at the latter, while the siphons extend into the surrounding water to maintain contact with the external environment (Shipway, Altamia, et al. 2019). A distinctive feature of shipworms is that the siphonal retractor muscles typically insert on the calcareous burrow lining rather than the shell valves, anchoring the posterior end of the animal to the burrow (Turner 1966).

Departures from this obligate wood‐based lifestyle are few. The giant shipworm *Kuphus polythalamius* represents an intermediate case, shifting from a wood‐associated early life stage to a sediment‐dwelling adult and relying on sulphur‐oxidising chemoautotrophic symbionts rather than the cellulolytic symbiosis typical of wood‐digesting shipworms (Distel et al. 2017; Shipway et al. 2018; Altamia et al. 2019). *Lithoredo abatanica*, however, represents a more radical departure: the only known shipworm not obligately associated with wood at any point in its life cycle, instead boring into and ingesting carbonate limestone within an oligohaline–freshwater river system (Shipway, Altamia, et al. 2019; Shipway, Rosenberg, et al. 2019).

Populations of *L. abatanica* form dense aggregations along the banks of the Abatan River (Antequera, Bohol, Philippines), where their closely spaced burrows extensively perforate and weaken limestone bedrock. These burrows form complex galleries that increase substrate heterogeneity and provide habitat for a range of associated taxa. In doing so, this species functions as a dominant ecosystem engineer, capable of modifying riverbed structure, promoting sediment production and influencing local biodiversity.

Despite the ecological significance of *L. abatanica*, its reproductive and larval biology remain unknown. Its known range is restricted to a short stretch of the Abatan River in a region that is difficult to access. As a result, few well‐documented observations are available. This restricted distribution also presents a fundamental ecological puzzle: how a population is maintained approximately 15 km upstream within a predominantly downstream flowing river system, where larval stages would be expected to experience downstream advection. Resolving this requires an understanding of life‐history strategy, particularly reproductive mode and dispersal potential.

Shipworms exhibit some of the most diverse and unusual reproductive strategies known among the Bivalvia (Shipway et al. 2020). While broadcast spawning is the most common mode of reproduction (15/18 known genera), several species internally fertilise, either via spermcasting or self‐fertilisation, and subsequently brood larvae (Treneman et al. 2026). This includes the genera *Lyrodus* and *Teredo*, and the seagrass‐boring species *Zachsia zenkewitschi*. In the latter case, internal fertilisation results from sperm cast by a harem of diminutive dwarf males retained in a specialised pouch near the female siphon (Shipway et al. 2016; Treneman et al. 2026). Additionally, at least four species, *Bankia gouldi* , *B. setacea* , *B. martensi* and *Nausitora fusticula* , have been reported to engage in pseudocopulation, a rare form of direct sperm transfer in which neighbouring individuals inseminate one another via their siphons (Huber 2010; Hiroki et al. 1994). In these species, pseudocopulation can involve complex interactions, including mate guarding, competitive siphon wrestling and even the active removal of rival spermatozoa (Shipway et al. 2020).

Whether this freshwater, rock‐boring species reproduces via broadcast spawning, spermcasting or a form of direct fertilisation has important implications for understanding its life history, dispersal potential, population structure and upstream persistence as well as the evolution of reproductive strategies within the Teredinidae. More broadly, resolving the life history of *L. abatanica* is important for placing this species within the still poorly understood ecological and evolutionary context of freshwater macrobioerosion. Here, we investigate the reproductive mode and life history strategy of *L. abatanica* using population size structure, siphon morphology and sperm morphometrics, and relate our findings to shipworm reproductive diversity and the persistence of a large freshwater macrobioeroder capable of reshaping riverbed structure, habitat complexity and associated biodiversity.

## 2 Methods

### 2.1 Collections

*Lithoredo abatanica* specimens were collected from the Abatan River (Antequera, Bohol, Philippines) as part of a Philippine Mollusk Symbiont (PMS) International Cooperative Biodiversity Group (ICBG) expedition in August 2018. This work was conducted in compliance with all required legal instruments and regulatory issuances under the Department of Agriculture‐Bureau of Fisheries and Aquatic Resources, Philippines (DA‐BFAR), under gratuitous permit 0140‐17. Opportunistic in situ photographs were taken using a Panasonic Lumix underwater digital camera to document individuals with their siphons extended from the limestone. Small rocks and boulders, typically around football size, were collected from the riverbed by snorkelling and freediving and brought to the riverbank for processing. A total of 70 individuals were subsequently extracted from limestone using a hammer and chisel, photographed and fixed ex situ. Full collection and deposition details, including type material, are provided in the original species description (Shipway, Altamia, et al. 2019; Table 1).

### 2.2 Microscopy

Specimens for anatomical and morphological examination were fixed in 4% formaldehyde solution freshly prepared from paraformaldehyde, washed, and dehydrated through a graded ethanol series (30%, 50% and 70%, 30 min per wash, ×2 washes) with final storage in 70% ethanol. Morphological features of soft tissues were imaged using the Keyence VHX‐6000 digital microscope (Osaka, Japan). Scanning electron microscopy (SEM) was used to examine the gonad of one mature specimen to confirm the presence of spermatozoa and obtain material for sperm morphometrics. This specimen was dehydrated through a complete ethanol series with a triple wash in absolute ethanol (30 min per wash), dissected, critical point dried using the Samdri PVT‐3D critical point dryer (Tousumis, Maryland, USA), mounted on a standard aluminium SEM stub and coated with platinum to a thickness of 5 nm using the Cressington 208 HR High Resolution Sputter Coater (Cressington Scientific Instruments, Hertfordshire, UK). Imaging was conducted using the Hitachi S‐4800 field‐emission scanning electron microscope.

### 2.3 Body and Burrow Measurements

Total body length was measured from the anterior margin of the shell valves to the posterior end of the siphons using ImageJ (Schneider et al. 2012) for all 70 extracted specimens. One intact empty burrow was measured along its full length using a ruler, from the posterior to anterior end. As shipworms occupy essentially the full length between the burrow aperture and active excavation face, intact empty burrows provide approximate records of the maximum length attained by their former occupants.

### 2.4 Comparative Dataset

To contextualise *L. abatanica* among freshwater rock‐boring macroinvertebrates, we assembled a comparative dataset from primary‐source records of extant, formally named species directly documented excavating lithified mineral substrates at demonstrably freshwater sites. Records involving unconsolidated sediment, firmground, hard clay, wood, shells, fossil traces or unidentified tracemakers were excluded. For each qualifying species, we extracted taxonomic identity, locality, substrate, evidence of active excavation, reported body size and reported boring dimensions (Table 1).

**TABLE 1:** Named extant freshwater macroinvertebrates documented excavating lithified rock.

Species	Higher taxon	Location	Substrate	Excavation evidence	Body size	Burrow dimensions	References
*Lithoredo abatanica*	Bivalvia: Teredinidae	Abatan River, Bohol, Philippines	Limestone; carbonate bedrock	Individuals occur within self‐produced, calcareously lined borings and ingest the excavated limestone	Total body length 5.46–105.4 mm; mean 40.3 ± 23.6 mm; *n* = 70. Measured from the anterior shell valves to the posterior siphons	One complete intact empty burrow measured > 200 mm. Qualitative in‐water observations indicated borings possibly exceeding 500 mm	Shipway, Altamia, et al. (2019); Shipway, Rosenberg, et al. (2019); present study
*Lignopholas fluminalis*	Bivalvia: Pholadidae	Kaladan River, Myanmar	Siltstone or aleurolite	Living individuals documented inside clavate borings assigned to *Gastrochaenolites anauchen*	NR numerically. Scaled specimens are illustrated in fig. 4A	NR numerically. Borings described as clavate and circular in cross‐section, expanding below the aperture	Bolotov et al. (2018)
*Balwantia soleniformis*	Bivalvia: Unionidae	Dhaleswari River, India	Friable sandstone and hard blue shale	Shells occurred in matching sandstone borings; worn anterior valve margins and corresponding shell and boring ridges were interpreted as evidence of mechanical excavation. Animals were also recovered by splitting shale rock	NR numerically in the sources examined. A modern specimen is illustrated with a 10 mm scale bar in Bolotov et al. (2024), fig. 4A	Four sandstone borings documented. Cross‐sectional height of one completed boring 89 mm; greatest breadth 46 mm; lower‐notch depth approximately 8 mm. Total longitudinal boring length NR	Godwin‐Austen (1919); Annandale (1919); Bolotov et al. (2024)
*Axarus festivus*	Insecta: Diptera: Chironomidae	Leavenworth County State Lake, Kansas, USA	Highly weathered Pennsylvanian shale	Larvae constructed burrows directly into exposed shale; isolated fourth‐instar larvae also constructed new shale burrows experimentally	NR	Total length mean 42 mm, range 37–53 mm; diameter mean 1.8 mm, range 1.5–2.4 mm; depth 18–29 mm; *n* = 40 burrows	Ferrington (1992)
*Languidipes lithophagus*	Insecta: Ephemeroptera: Polymitarcyidae	Bago River, Myanmar	Siltstone or aleurolite	Living nymphs documented within self‐produced borings; excavation performed using enlarged mandibular tusks	Holotype 14.3 mm; paratypes 11.3–17.9 mm, *N* = 15	A ‘typical’ boring measured 20.1 mm in total length and 4.4 mm in maximum diameter. This is not identified as a mean or maximum	Bolotov et al. (2022)
*Povilla adusta*	Insecta: Ephemeroptera: Polymitarcyidae	Lake Kainji, Nigeria	Sandstone rock	Nymphs explicitly reported burrowing into sandstone exposed by wave action; also wooden boats and natural wood substrates	Lake Kainji final‐instar males 7.4–11.3 mm, mean 10.0 ± 1.3 mm; females 13.0–22.0 mm, mean 17.0 ± 2.0 mm	NR for sandstone borings	Bidwell (1979)

*Note:* Only formally named species for which primary sources directly document active excavation of lithified mineral substrates at demonstrably freshwater sites are included. Body‐size and boring measurements are reported exactly as provided in the original sources.

Abbreviation: NR, not reported.

### 2.5 Reproductive Mode

Spermatozoa were imaged and measured in ImageJ (Schneider et al. 2012), with acrosomal apparatus length and sperm body length quantified for 100 individual cells from a single mature individual. The ratio of acrosomal apparatus length to sperm body length was calculated for each spermatozoon and used as an indicator of fertilisation mode, following the morphometric framework established by Popham (1974), which demonstrated that higher ratios are characteristic of externally fertilising species. The mean ratio and associated variability were used for comparison with published values. As suitable SEM material was limited, sperm morphometrics were obtained from a single mature individual and are therefore interpreted here as indicative rather than population‐level estimates.

### 2.6 Use of AI‐Assisted Editing Tools

Minor editorial revisions to improve spelling, grammar and clarity were supported using ChatGPT version 5.4. No AI tools were used for data analysis, interpretation or generation of scientific content.

## 3 Results

**FIGURE 1:**
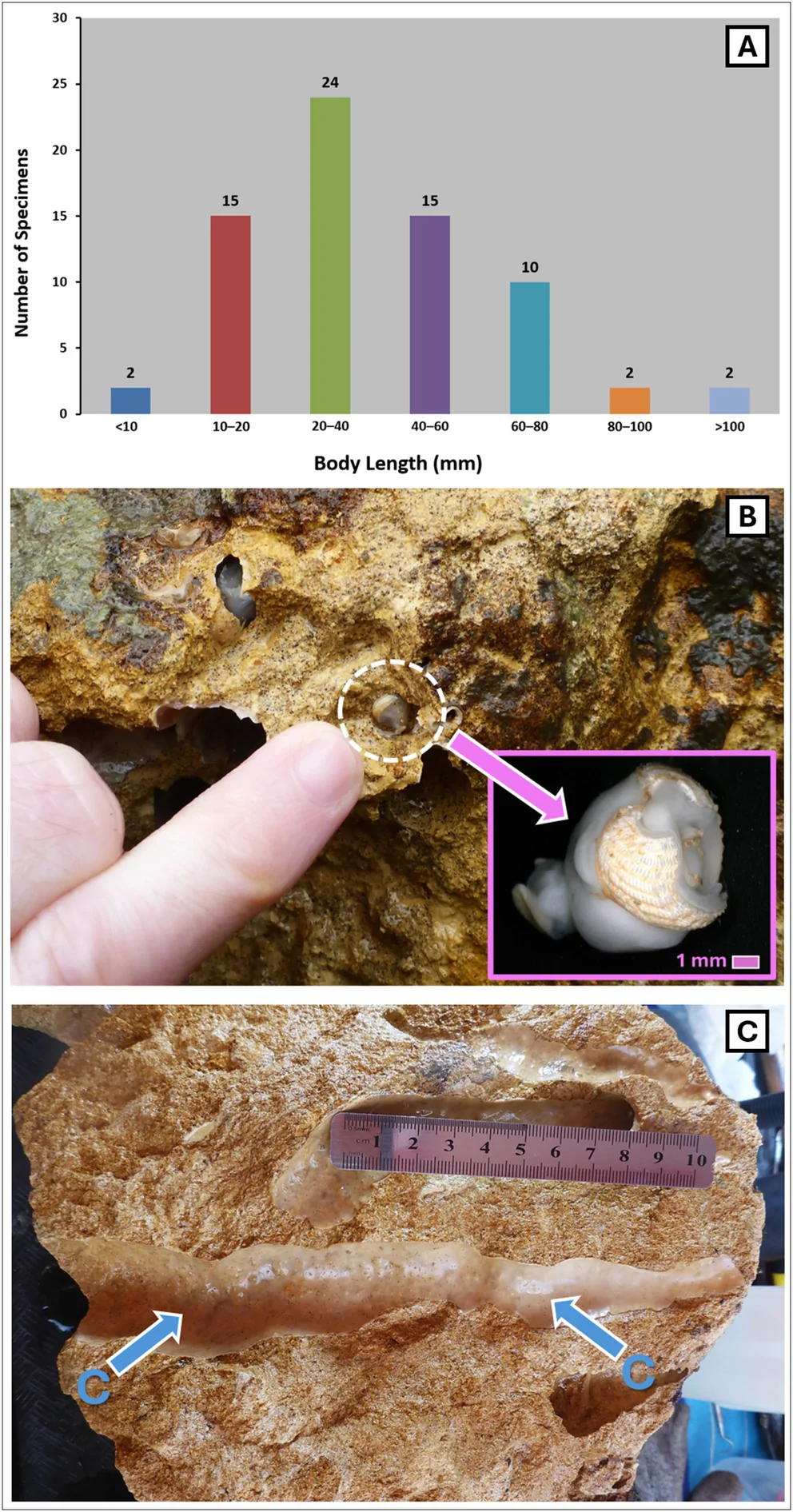
Size distribution and evidence of recent recruitment in *Lithoredo abatanica*. (A) Distribution of specimen body lengths grouped into discrete size classes, showing the number of specimens within each body‐length bin. Numbers above bars represent observed specimen counts; overall mean ± SD body length was 40.3 ± 23.6 mm (*n* = 70). (B) Specimen measuring ~5 mm in total body length. (C) Intact empty burrow > 200 mm long; because shipworms occupy essentially the full distance between the burrow aperture and excavation face, this provides an approximate record of former body length. C, calcareous tube. Scale bar in panel B inset = 1 mm; ruler in C is graduated in centimetres.

Total body lengths (defined as anterior of the shell valve to the posterior siphon) were measured for all collected specimens (Figure 1A). The smallest and largest specimens measured 5.46 and 105.4 mm (Figure 1B), respectively, with an average total body length of 40.3 mm. One complete intact empty burrow exceeded 200 mm in length (Figure 1C). Because shipworms occupy essentially the full distance between the posterior aperture and the blind anterior excavation face, this burrow provides an approximate record of a former occupant larger than any individual successfully extracted during the collection. Body length measurements, arranged into size bins (Figure 1A), revealed two individuals with total body lengths < 10 mm. These tiny individuals (Figure 1B) indicate recent settlement onto limestone and subsequent metamorphosis.

The comparative dataset comprised six named extant freshwater macroinvertebrates directly documented excavating lithified rock: three bivalves, two mayflies and one chironomid (Table 1). Among the named freshwater rock‐borers for which longitudinal boring measurements are available, *L. abatanica* has the greatest documented boring extent, with one directly measured intact burrow exceeding 200 mm and qualitative in‐water observations indicating burrows possibly exceeding 500 mm.

**FIGURE 2:**
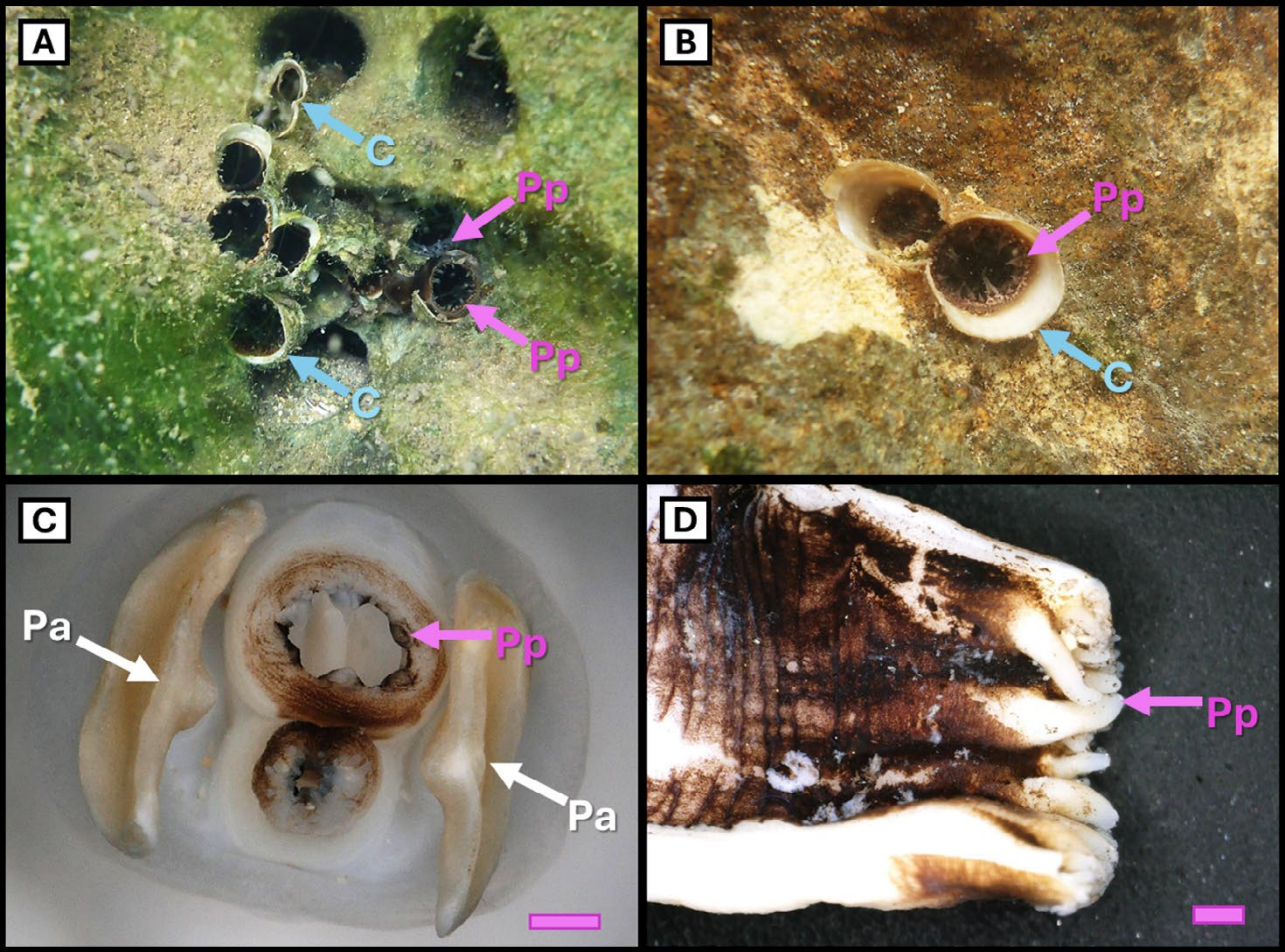
Siphonal and calcareous tube morphology likely precludes pseudocopulation in *Lithoredo abatanica*. (A) Multiple individuals of *L. abatanica* clustered in limestone with siphons extended. (B) A single *L. abatanica* individual showing a figure‐of‐eight calcareous tube protruding beyond fully extended siphons. (C) Posterior end‐on view of *L. abatanica* specimen removed from limestone, with arrow highlighting papillae on the incurrent siphon. (D) Dissected incurrent siphon. C, calcareous tube; Pa, pallet; Pp, papillae. Scale bars = 500 μm.

Examination of *Lithoredo abatanica* in situ revealed specimens occurring in dense aggregations within limestone, with siphons from multiple individuals occurring in close proximity (Figure 2A). The calcareous burrow linings, which form an extension of the calcareous tube, projected beyond the limestone surface and around the siphonal openings in an almost complete figure‐of‐eight arrangement (Figure 2A,B). We did not observe the siphons extending beyond this calcareous extension of the burrow lining. The siphons were fused along most of their length; only the distal portion was unfused, and this portion remained enclosed within the burrow lining (Figure 2B). Dense papillae were also present, particularly on the incurrent siphon (Figure 2C,D).

**FIGURE 3:**
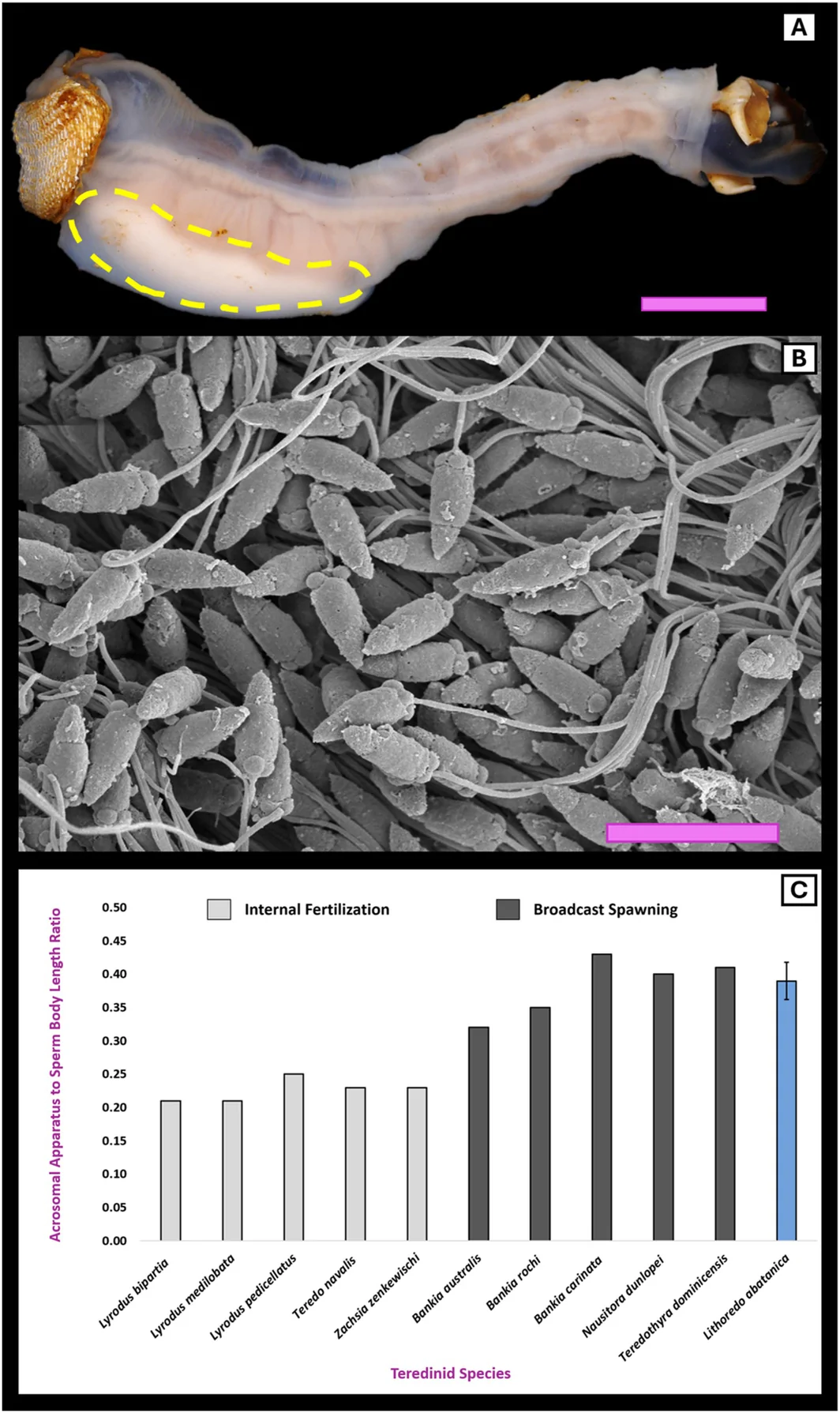
*Lithoredo abatanica* sperm morphology is consistent with externally fertilising, broadcast spawning teredinids. (A) Mature specimen featuring a full gonad (yellow dashed outline). (B) Scanning electron micrograph of *L. abatanica* spermatozoa. (C) Mean acrosomal apparatus‐to‐sperm body length ratio for *L. abatanica* compared with published teredinid values. The *L. abatanica* value represents the mean ± SD of 100 spermatozoa measured from one mature individual. Comparator values are from Popham (1974) and Shipway et al. (2014), the latter for *Teredothyra dominicensis*. Scale bars in A and B = 10 mm and 5 μm respectively.

Several larger individuals of *L. abatanica* featured well‐developed gonads (Figure 3A). Examination of spermatozoa (*n* = 100) from one mature individual showed a mean ratio of acrosomal apparatus length to sperm body length of 0.39 ± 0.028 SD (Figure 3B,C). In Popham's (1974) comparison of nine teredinid species, this ratio ranged from 0.21 to 0.25 in internally fertilising taxa and from 0.32 to 0.43 in externally fertilising taxa, with no overlap between categories; the value observed for *L. abatanica* falls within the externally fertilising range.

## 4 Discussion

The habitat, morphology and systematics of *Lithoredo abatanica* are well documented for a recently described bivalve, but its reproductive mode and larval ecology have remained uncharacterised; here, we provide the first evidence‐based assessment of these traits. The comparative dataset assembled in this study further establishes *L. abatanica* as the largest known freshwater rock‐boring macrobioeroder. Its exceptional body and burrow dimensions, together with the dense aggregations observed in situ, indicate considerable capacity for local rock breakdown and habitat modification within the Abatan River system.

Morphometric evidence is consistent with recent recruitment and ongoing reproduction in the Abatan River population. Very small individuals (< 10 mm total body length) embedded within limestone are consistent with recent larval settlement and metamorphosis (Figure 1A,B). The co‐occurrence of these likely recently settled individuals with larger specimens bearing fully developed gonads (Figure 3A) is consistent with ongoing reproduction and recent recruitment at the time of collection (August 2018). However, as these observations derive from a single population and collection event, they do not resolve spatial or temporal variation in reproductive activity.

Although the largest specimen collected in this study measured approximately 10 cm in total body length, substantially larger (> 20 cm) empty limestone burrows were observed (Figure 1C). As shipworms occupy essentially the full distance between the posterior aperture and the blind anterior excavation face, these provide approximate records of the maximum body length attained by their former occupants. Our sampling was necessarily biased towards smaller rocks and boulders that could be collected by freediving and transported to the riverbank for processing. Larger bedrock‐embedded individuals were inaccessible using these methods. Observations made by the author (J.R.S.) during a SCUBA dive in the main riverbed further revealed the presence of extremely large individuals, conservatively estimated to exceed 50 cm in length, which proved exceptionally difficult to extract from the surrounding limestone in flowing water. These observations strongly suggest that the collected material considerably underestimated the maximum body size of *L. abatanica*.

Recent global analyses of shipworm growth rates and maximum sizes place *L. abatanica* toward the upper extreme of body size within the Teredinidae, while the metre‐scale lengths attained by other teredinids demonstrate that the exceptional dimensions documented here are biologically plausible within the family (Poon et al. 2025). The comparative dataset presented in Table 1 establishes *L. abatanica* as the largest currently known freshwater rock‐boring macrobioeroder, exceeding 100 mm in body length and producing burrows longer than 200 mm, with in‐water observations indicating burrows potentially exceeding 500 mm. This exceptional individual scale gives *L. abatanica* considerable capacity to modify limestone habitat within the Abatan River through rock fragmentation, increased substrate porosity, sediment production and the generation of structurally complex habitat for associated biota, with potential consequences for riverbed stability, channel morphology and substrate turnover that remain to be quantified (Shipway, Rosenberg, et al. 2019). Extensive boring of lithified rock appears uncommon in freshwater (Bolotov et al. 2018, 2022), although it is also likely under‐recorded. Future work should determine whether *Lithoredo* occurs in other river systems through targeted biological surveys combined with geological mapping of suitable carbonate substrates and submerged hardgrounds; collaboration with a geologist would help identify lithologies, map habitat continuity and define the distribution of potentially suitable rock‐boring habitat. Excavation rates could also be measured experimentally by adapting approaches used in other marine wood‐borers, in which faecal or frass production provides a practical proxy for substrate ingestion and degradation; combined with repeated body and burrow measurements, these methods could quantify how rapidly *L. abatanica* excavates rock and grows (Willer et al. 2023; Martin et al. 2025).

Our in situ observations provide the first record of *L. abatanica* exhibiting natural behaviour within limestone (Figure 2), offering critical insight into its life‐history strategy and reproductive mode. The calcareous tube extends beyond the siphons and forms a rigid figure‐of‐eight structure that physically separates the incurrent and excurrent siphons (Figure 2B). This tube architecture, combined with short siphons that extend only minimally into the water column and are joined along nearly their entire length, makes pseudocopulation, a form of internal fertilisation documented in only a small number of shipworm species (Hiroki et al. 1994; Shipway et al. 2020), anatomically very unlikely in *L. abatanica*. Moreover, the incurrent siphon, which functions as the sperm‐receiving structure in pseudocopulating shipworms, is lined with dense internal papillae (Figure 2). Dense papillae on the incurrent siphon may further reduce the feasibility of direct siphonal coupling, although the principal constraint appears to be the fused siphons and protruding figure‐of‐eight calcareous lining.

Although seemingly maladapted for pseudocopulation, the short, truncated siphons of *L. abatanica* may represent an adaptive response to life in a fast‐flowing freshwater environment subject to seasonal flooding and debris transport. Analogous selective pressures have been documented in other sessile invertebrates. For example, intertidal barnacles from wave‐exposed environments possess shorter, stouter and more massive penises than conspecifics from sheltered habitats, reflecting mechanical optimisation under high hydrodynamic stress (Neufeld and Palmer 2008). By analogy, reduced siphon length in *L. abatanica* may minimise damage or dislodgement in turbulent river flow while maintaining essential respiratory and feeding functions. While the original taxonomic description of *L. abatanica* detailed siphonal morphology (Shipway, Altamia, et al. 2019), reproductive mode was not considered in relation to these features. Our findings demonstrate that features of siphonal and calcareous tube morphology are not merely taxonomic characters but are intimately linked to reproductive strategy and life history.

These anatomical observations, together with the absence of retained larvae in material examined during the original species description, are consistent with reproduction via broadcast spawning with external fertilisation. This inference is further supported by sperm morphometrics. In Popham's (1974) comparison of nine teredinid species, the ratio of acrosomal apparatus length to sperm body length ranged from 0.21 to 0.25 in internally fertilising taxa and from 0.32 to 0.43 in externally fertilising taxa, with the latter identified by Popham as the metric showing the strongest correlation with fertilisation mode. The value observed for *L. abatanica* (0.39) falls within the externally fertilising range and closely matches values reported for broadcast‐spawning species such as *Teredothyra dominicensis* (Shipway et al. 2014; Figure 3). Popham (1974) proposed that the smaller acrosomal apparatus of internally fertilising teredinids reflects the greater efficiency of fertilisation within the mantle cavity, where sperm are more likely to encounter ova and acrosomal products are less subject to dilution than in seawater. In contrast, larger acrosomes and longer acrosomal rods may compensate for lower encounter efficiency and greater dilution of lytic material during external fertilisation. Together, siphonal morphology and sperm morphometrics provide supporting evidence for broadcast spawning as the likely reproductive mode in this species. This inference is also consistent with phylogenetic placement: *Lithoredo* diverges early in shipworm evolution, and lineages branching prior to the divergence of *Bankia* are broadcast spawners, whereas the internally fertilising, larval‐brooding and pseudocopulating taxa appear to have arisen more recently (Distel et al. 2011; Li et al. 2022; Treneman et al. 2026). While this result is based on a single mature individual and should therefore be treated as supportive rather than definitive in isolation, it is consistent with the phylogenetic placement of *L. abatanica* and, together with the absence of brooded larvae or brooding modification of the parental gill and the morphology of the siphons and calcareous tube, strengthens the inference that the species is a broadcast spawner.

Broadcast‐spawning teredinids feature a planktotrophic larval phase, which can persist for up to 4 weeks (Culliney 1975; MacIntosh et al. 2014). In a freshwater riverine system, this raises a fundamental question: how do larvae disperse and successfully settle up to 15 km upstream of the river mouth, where populations of *L. abatanica* have been documented in the Abatan River (Shipway, Altamia, et al. 2019)? This problem closely reflects the ‘drift paradox’, whereby downstream transport should progressively deplete upstream populations unless offset by retention or upstream return processes; Müller (1982) described the relevant colonisation dynamics, while later theoretical treatments formalised the concept more explicitly (e.g., Pachepsky et al. 2005). More broadly, work on the drift paradox has shown that persistence in advective systems can also be promoted by local retention, benthic refugia and other mechanisms that reduce net downstream loss, rather than requiring large‐scale upstream compensatory movement alone (Pachepsky et al. 2005). Plausible mechanisms include local retention of larvae in low‐velocity refugia such as boundary layers, eddies and cavities within the limestone created by adult boring, or passive upstream transport within an intruding salt wedge, which could carry larvae in suspension (Gibson et al. 2001; Allgayer et al. 2021). The complex burrow network may also shelter newly settled juveniles from strong currents and reduce their subsequent displacement. Spawning may also be timed to coincide with low‐flow conditions during the dry season, when reduced discharge could lessen downstream advection and potentially allow deeper saline intrusion; analogous hydrologically structured spawning windows are known in other riverine and estuarine taxa (Tyler et al. 2021). In addition, the nutritional ecology of *L. abatanica* may play an important role in structuring its distribution. Although the species ingests limestone, its true nutritional sources remain unresolved and may include suspended particulate material, biofilms, chemoautotrophic symbiont‐mediated nutrition akin to the sulphur‐oxidising endosymbiosis described in *Kuphus polythalamius* (Distel et al. 2017), or some combination of these rather than the rock itself as a direct food source (Shipway, Altamia, et al. 2019). It also remains unclear whether suitable carbonate habitat is continuous along the river, or whether *L. abatanica* is restricted to areas where substrate availability, low salinity, flow conditions and food resources coincide. Such a combination of hydrodynamic and environmental constraints may help explain the tendency of populations to persist upstream despite the expectation of downstream larval transport. More broadly still, *L. abatanica* may provide an unusual and informative test case for extending the drift‐paradox framework to a freshwater broadcast‐spawning benthic invertebrate with a prolonged larval phase.

The combination of hydrodynamic constraints, potential retention mechanisms and unresolved nutritional ecology outlined above suggests that the life history of *L. abatanica* may be defined not only by its transition from wood to stone, but by a potentially complex interplay of hydrodynamic strategy, habitat distribution, and nutritional or symbiotic ecology that promotes upstream persistence. Resolving these processes requires targeted larval sampling together with hydrodynamic modelling, population connectivity analyses and direct investigation of the species' nutritional and symbiotic strategy and is central to understanding dispersal, biogeography, and conservation status in this globally unusual freshwater bivalve.

In summary, we provide multiple independent lines of evidence indicating that *L. abatanica* is a broadcast‐spawning shipworm with external fertilisation and a planktotrophic larval phase and establish it as the largest known freshwater macrobioeroder. This combination of traits underscores the globally unique ecological role of this species and highlights the need for further work on larval ecology, dispersal mechanisms and population structure. Future studies integrating larval sampling, hydrodynamic modelling and genetic connectivity analyses will be critical for understanding how this remarkable bivalve shapes freshwater ecosystems and how vulnerable it may be to environmental change. Together, these findings provide the first detailed account of the life history and reproductive strategy of *L. abatanica*—a globally unique freshwater ecosystem engineer.

## 5 Conclusion

This study provides the first evidence‐based assessment of the reproductive biology and life history of *Lithoredo abatanica*, a freshwater rock‐boring shipworm with a highly unusual ecology among bivalves. By integrating population size structure, in situ observations, siphonal morphology and sperm morphometrics, we infer that *L. abatanica* reproduces via broadcast spawning with external fertilisation and a planktotrophic larval phase. These findings place *L. abatanica* firmly within the reproductive spectrum of other basal teredinids, despite its highly divergent habitat and ecology.

At the same time, our findings raise important unresolved questions. In particular, the mechanisms by which planktotrophic larvae disperse and settle far upstream in a freshwater river system remain unknown, with implications for population connectivity, biogeography and conservation. Addressing these questions will require targeted studies of larval ecology, symbiosis and nutrition, hydrodynamics and genetic structure. Together, this work establishes a foundation for future research on the biology, ecology and conservation of one of the most unusual bivalves known, and underscores the need to consider freshwater systems in broader discussions of bioerosion and ecosystem engineering.

## Author Contributions

**J. Reuben Shipway:** conceptualization (equal), data curation (lead), formal analysis (lead), investigation (lead), methodology (lead), writing – original draft (lead), writing – review and editing (lead). **Daniel L. Distel:** conceptualization (supporting), funding acquisition (lead), resources (lead), writing – review and editing (supporting).

## Funding

The research reported in this publication was supported by Fogarty International Center of the National Institutes of Health Award U19TW008163 (D.L.D.) and National Science Foundation Award IOS1442759 (D.L.D.).

## Conflicts of Interest

The authors declare no conflicts of interest.

## Data Availability

The data supporting the findings of this study have been deposited in Figshare and will be made publicly available upon publication at the existing DOI: https://doi.org/10.6084/m9.figshare.32488425.
